# Epidemiological comparison of international travel-associated and domestically acquired *Campylobacter*, *Salmonella*, and *Shigella* infections reported to Foodborne Diseases Active Surveillance Network (FoodNet), 2010–2019

**DOI:** 10.1016/j.tmaid.2026.103022

**Published:** 2026-09-10

**Authors:** Hazel J. Shah, Kristina M. Angelo, Sophia Wozny, Sarah Lathrop, Sean Buuck, Melissa Tobin-Dangelo, Rosalie Trevejo, Kerianne Engesser, Elaine Scallan Walter, Marcy McMillan, Christina Turner, Katie Wymore, Robin P Pendley Louis, Daniel L. Weller

**Affiliations:** a Division of Foodborne, Waterborne, and Environmental Disease, U.S. Centers for Disease Control and Prevention, Atlanta, GA, USA; b Emerging Infections Program, Maryland Department of Health, Baltimore, MD, USA; c University of New Mexico, Albuquerque, NM, USA; d Minnesota Department of Health, St. Paul, MN, USA; e Georgia Department of Public Health, Acute Disease Epidemiology Section, Atlanta, GA, USA; f Public Health Division, Oregon Health Authority, Portland, OR, USA; g New York State Department of Health, Albany, NY, USA; h Colorado School of Public Health, University of Colorado Anschutz Medical Campus, Aurora, CO, USA; i Tennessee Department of Health, Nashville, TN, USA; j Connecticut Department of Public Health, Hartford, CT, USA; k California Emerging Infections Program, Oakland, CA, USA

**Keywords:** Travel-associated illness, Enteric illness, FoodNet, Travel, Diarrhea, Gastroenteritis

## Abstract

**Background:**

A 2004–2009 analysis found that 13% of enteric illnesses reported in the United States were associated with international travel, with >90% caused by *Campylobacter*, *Salmonella*, or *Shigella*. This analysis characterizes the epidemiology of travel-associated campylobacteriosis, salmonellosis, and shigellosis in the FoodNet catchment during 2010–2019.

**Methods:**

We analyzed FoodNet surveillance data for laboratory-confirmed infections during 2010–2019. Characteristics of travel-associated and domestically acquired infections were compared between world regions using incidence rate ratios (IRRs) and counterfactual random forest (CFRF) methods.

**Results:**

During 2010–2019, 61,397 *Campylobacter*, 63,823 *Salmonella*, and 19,141 *Shigella* infections were reported. Among people with known travel status, 16%, 11%, and 15% of *Campylobacter*, *Salmonella*, and *Shigella* infections were travel-associated. Incidence of travel-associated campylobacteriosis, salmonellosis and shigellosis increased from 2004 to 2009 to 2010–2014 and from 2010 to 2014 to 2015–2019. More travelers reported visiting Latin America and the Caribbean (2010–2014: 44%; 2015–2019: 45%) but illness incidence was highest among travelers to Africa (2010–2014: 49.8 per 100,000; 2015–2019: 57.8 per 100,000). Demographic and clinical characteristics of people with travel-associated versus domestically acquired infections differed consistently across the three illnesses. Odds of reporting international travel were higher among urban versus rural residents, among people with mild versus severe illness, and among people diagnosed using culture-independent versus culture-based tests.

**Conclusions:**

These findings underscore the importance of collecting travel history during clinical encounters and enteric disease investigations. Findings can help inform public health messaging and enteric disease prevention strategies provided to international travelers.

## 1. Introduction

Diarrhea is the most common travel-associated illness among international travelers, with attack rates ranging from 30 to 70% depending on destination, season, and other factors [1,2]. Bacterial enteric pathogens cause approximately 80% of travel-associated diarrhea [3], most frequently enterotoxigenic and enteropathogenic *Escherichia coli* [4,5]. *Campylobacter*, nontyphoidal *Salmonella* (NTS) and *Shigella*, account for approximately 10–20% of illnesses [6]. Infection with these pathogens can result from ingestion of contaminated food or water; poor hygiene and sanitation practices during food preparation at travel destinations can also increase the risk of infection [3].

The Foodborne Diseases Active Surveillance Network (FoodNet) has collected travel history for U.S. residents with laboratory-confirmed enteric infections since the mid-1990s. Analysis of FoodNet data from 2004 to 2009 found that 13% of reported enteric infections were associated with international travel [7]. *Campylobacter*, NTS, and *Shigella* were the most common pathogens detected among travelers, but not all pathogens (including some *Escherichia coli* pathotypes) are collected through FoodNet surveillance [7].

Since 2009, there have been substantial changes in diagnostic practices, surveillance, and travel behavior, which may affect which illnesses are diagnosed and reported, and thereby, change our understanding of travel-associated enteric infections [8–12]. Notably, the increasing use of culture independent diagnostic tests (CIDTs) in the 2010s has facilitated ascertainment of illnesses that would have previously gone undiagnosed and has been associated with sharp increases in reported enteric illness incidence, altering long-standing surveillance trends and complicating comparisons over time [13,14]. The advent of CIDTs may have also facilitated detection of travel-associated illnesses that would have otherwise been undiagnosed [15].

The aim of this analysis is to improve our understanding of international travel-associated enteric illnesses reported to FoodNet focusing on *Campylobacter,* NTS *Salmonella,* typhoidal *Salmonella*(TS) and *Shigella* infections. Findings from this analysis may be used to supplement existing enteric disease prevention recommendations provided to international travelers.

## 2. Methods

### 2.1. Data sources

Data were obtained from FoodNet on laboratory-confirmed *Campylobacter*, all *Salmonella*, and *Shigella* infections reported during 2010–2019. FoodNet is a collaboration of the U.S. Centers for Disease Control and Prevention (CDC), 10 state health departments, the U.S. Department of Agriculture’s Food Safety and Inspection Service (USDA-FSIS), and the Food and Drug Administration (FDA) that conducts active, population-based surveillance for laboratory-diagnosed infections caused by eight pathogens. During 2004–2019, FoodNet’s catchment included Connecticut, Georgia, Maryland, Minnesota, New Mexico, Oregon, Tennessee, and selected counties in California, Colorado, and New York, representing approximately 15% of the U.S. population [14].

To calculate disease incidence among travelers to various areas, travel destination data from the U.S. Department of Commerce’s Survey of International Air Travelers (SIAT) were obtained. U.S. adult (≥18 years of age) residents flying on select airlines, representing 95% of the United States’ international air traffic, were randomly selected to participate in SIAT; SIAT excludes travel to Canada [16].

### 2.2. Definitions

A case was considered travel-associated if international travel was reported during the 7 days preceding illness onset for persons with a *Campylobacter*, NTS, and *Shigella* infection, and the 30 days preceding onset for those with a typhoidal and paratyphoidal *Salmonella* infection [14]. A case was considered domestically acquired if international travel was not reported during these periods. Persons with unknown travel status were excluded.

For many years, FoodNet only collected data on culture-diagnosed infections. Beginning in 2009, FoodNet sites started transmitting data on cases diagnosed using culture-independent methods. Testing practices varied by pathogen and year, with many laboratories adopting culture-independent methods in 2015 following FDA-approval of the first gastrointestinal syndromic panels [17]. Following a culture-independent diagnosis, reflex culture could be attempted by the clinical, local, or state laboratory. Following a culture-based diagnosis or a CIDT-diagnosis with a positive reflex culture speciation and subtyping may have been performed. In the United States, most CIDTs detect *Salmonella enterica* spp. (which includes both NTS and TS). Reflex culture or a typhoidal *Salmonella* specific CIDT panel is necessary for identification.

Travel destinations were categorized into 6 world regions as defined by the United Nations Statistics Division as done in other FoodNet travel analyses: Africa; Asia; Europe; Latin America (including Mexico) and the Caribbean; North America (Bermuda, Canada, and Greenland); and Oceania [18]. If the traveler visited multiple regions, they were categorized as multi-region travelers and excluded from regional analyses.

### 2.3. Analysis

*Campylobacter*, *Salmonella*, and *Shigella* infections reported to FoodNet during 2010–2019 were described and then compared across two, 5-year time periods (2010–2014 versus 2015–2019) to identify changes over time using incidence rate ratios (IRRs). These comparison periods were selected to broadly align with major changes in diagnostic testing and surveillance during the study period, including FDA approval of the first gastrointestinal syndromic panels with targets for *Campylobacter*, *Salmonella*, and *Shigella*, increasing use of CIDTs, and changes to FoodNet case definitions, rather than to evaluate the effect of any individual change [17]. *Salmonella* serotypes were ranked separately by the number of travel-associated and domestically acquired infections associated with them; serotype differences between the percent of travel-associated and domestically acquired illnesses attributed to the ten most frequently reported travel-associated serotypes during 2010–2019 were evaluated using chi-square (*P* < 0.05).

We calculated incidence rates (IRs) of travel-associated infections among residents of FoodNet catchment areas, by world region, per 100,000 persons. To align with the SIAT restrictions, travel-associated infections among individuals aged ≤17 years were excluded from the incidence analyses only. All other analyses included all available data, including travel-associated infections among individuals aged ≤17 years. We did not calculate a region-specific incidence rate for North America because the frequencies were too small and the lack of SIAT data for those traveling to Canada. We used SAS software (version 9.4: SAS Institute, Cary, North Carolina) for descriptive analyses.

To compare the epidemiology of travel-associated and domestically acquired infections while accounting for correlated covariates, we adapted counterfactual random forest (CFRF) analysis [19] in R version 4.40. CFRFestimates adjusted odds ratios by constructing counterfactual contrasts under observed covariate distributions. Models included demographic characteristics, hospitalization status, symptoms, testing practices, rurality, region, and year. Rurality was a binary variable reflecting if the county of residence was classified as urban or rural using the USDA Rural-Urban Continuum Codes (RUCC; https://www.ers.usda.gov/data-products/rural-urban-continuum-codes). RUCC values 1–3 were classified as urban and values 4–9 were classified as rural. To maximize sample size, missing values were treated as “Not Reported,” rather than dropping missing data. Separate CFRFs were fit for each illness with the outcome if the illness was travel-associated or not.

## 3. Results

During 2010–2019, 61,397 *Campylobacter*, 63,823 *Salmonella,* and 19,141 *Shigella* infections were reported to FoodNet. Among persons with known travel status, 16% (10,015/61,397) of *Campylobacter*, 11% (6804/63,823) of *Salmonella*, and 15% (2899/19,141) of *Shigella* infections were travel-associated (Table 1).

During both periods considered, more travelers reported visiting Latin America and the Caribbean (2010–2014: 44%; 2015–2019: 45%), but the incidence of illness was highest among travelers to Africa (2010–2014: 49.8 per 100,000; 2015–2019: 57.8 per 100,000; Table 2).

Travelers’ race and ethnicity strongly correlated with travel destinations. Most Asian travelers (80%) traveled to Asia; most Black travelers visited either Africa (43%) or Latin America and the Caribbean (40%); most Hispanic travelers traveled to Latin America and the Caribbean (89%).

### 3.1. Campylobacter

**Fig. 1.**
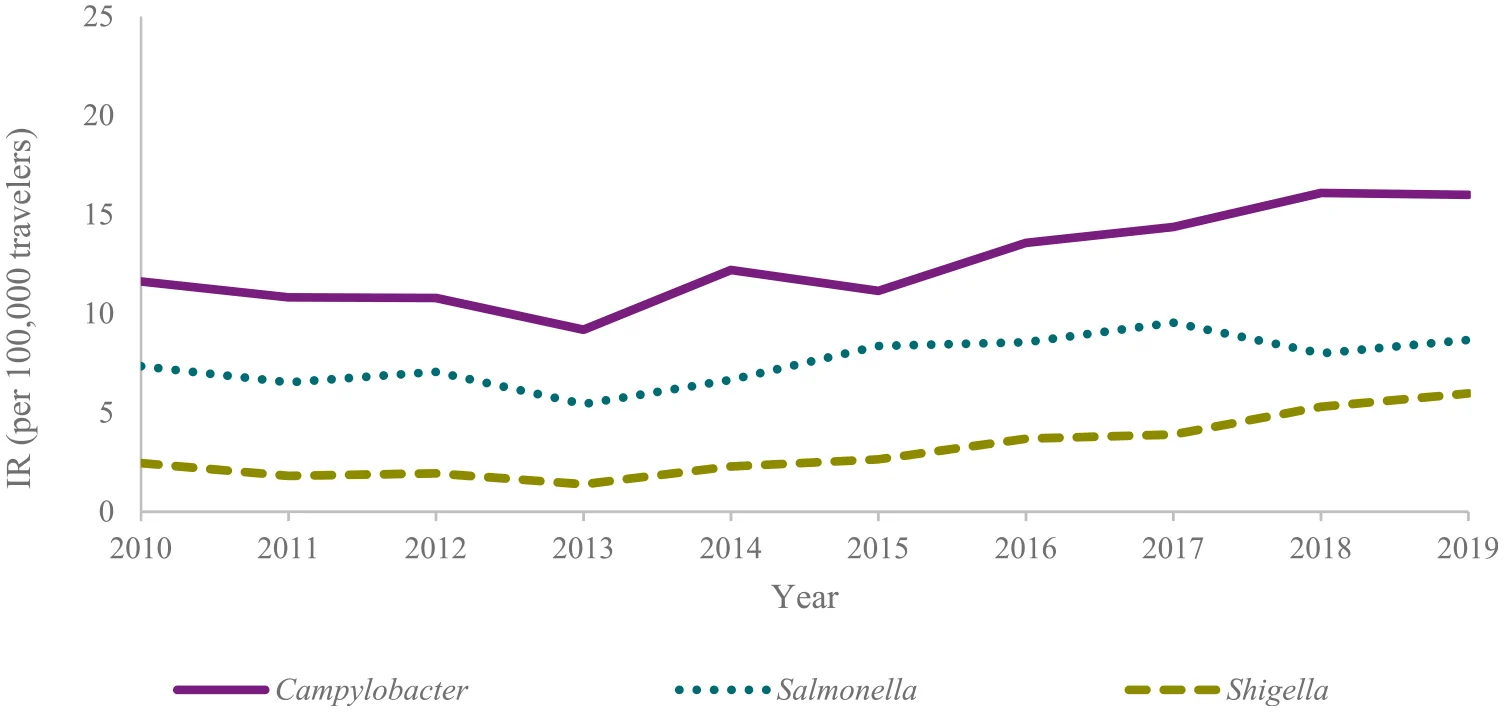
Incidence rate (IR) of international travel-associated infections among persons aged 18 and over, by pathogen and year — FoodNet, 2010–2019.

**Fig. 2.**
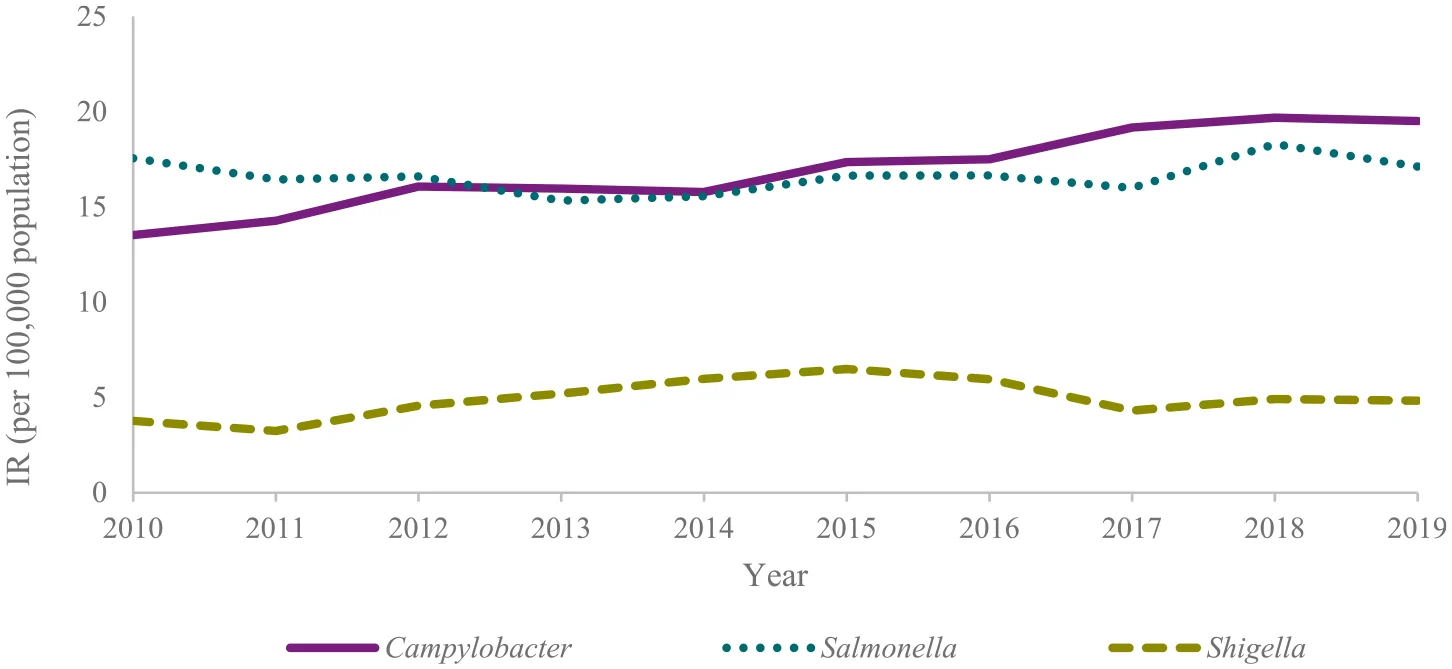
Incidence rate (IR) of all infections reported to FoodNet regardless of age or travel-status, by pathogen and year — FoodNet, 2010–2019.

**Fig. 3.**
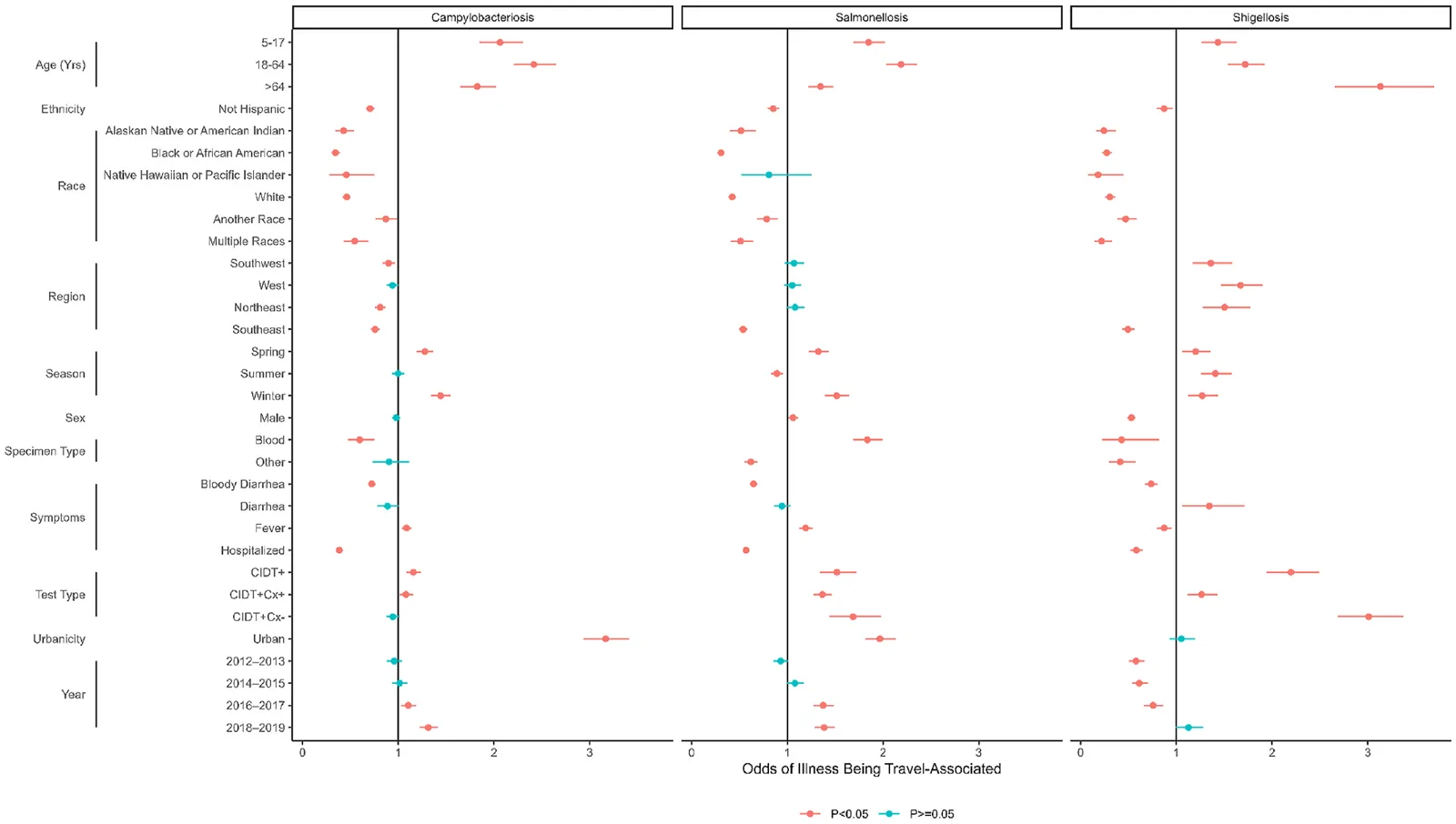
Results of the counterfactual random forest where the outcome was if the illness was domestically acquired (reference) or travel-associated; bars indicate 95% confidence interval for the odds ratio. Results are not reported for persons <5, Asian persons, Hispanic persons, persons residing in the Mid-Atlantic states or rural counties, persons who became ill in Autumn or in 2010–2012, female persons, people diagnosed using stool specimens or culture-based (Cx) methods only, person not reporting diarrhea, bloody diarrhea, or fever, and persons that were not hospitalized since these were the reference levels. CIDT = culture independent diagnostic test. CIDT+ is CIDT diagnosis only; CIDT + Cx+ is CIDT followed by positive reflex culture; CIDT + Cx-is CIDT followed by negative reflex culture.

The incidence of travel-associated and domestically acquired campylobacteriosis among travelers aged ≥18 years increased from 2010 through 2019 (Figs. 1 and 2; Tables S1 and S2). The incidence of travel-associated campylobacteriosis was 32% higher during 2015–2019 compared to 2010–2014 (Table 2). According to CFRF, the odds of any illness being travel-associated was higher during 2016–2017 (Odds Ratio [OR] = 1.1; 95% Confidence Interval [CI] = 1.0, 1.2) and 2018–2019 (OR = 1.3; 95% CI = 1.2, 1.4) compared to 2010–2011 (Fig. 3).

Nearly all persons with travel-associated *Campylobacter* during 2015–2019 traveled to a single region (9713/9828, 99%). During 2010–2014 and 2015–2019, most travel-associated illnesses were linked to Latin America and the Caribbean followed by Asia and Europe, although incidence was highest among travelers to Africa followed by Asia, and Latin America and the Caribbean (Table 2). Incidence was lowest among travelers to Oceania and Europe. The incidence of *Campylobacter* infections increased significantly from 2010 to 2014 to 2015–2019 among travelers visiting Asia (IRR, 1.7 [95% CI, 1.5–1.8]), Latin America and the Caribbean (IRR, 1.2 [95% CI, 1.2–1.3]), and Europe (IRR, 1.2 [95% CI, 1.1–1.3]) (Table 2).

Of the 4112 speciated travel-associated *Campylobacter* infections, the majority were caused by *C. jejuni* (n = 3569). *C. coli* (n = 503), *C. upsaliensis* (n = 15), *C. lari* (n = 11), *C. fetus* (n = 7), *C. consisus* (n = 4), *C. hyointestinalis* (n = 2), *C. curvus* (n = 1) were also linked with travel-associated *Campylobacter* infection.

### 3.2. Salmonella

Although interannual variation in the incidence of travel-associated and domestically acquired salmonellosis appeared modest during 2010–2019 (Figs. 1 and 2), the average incidence of travel-associated infections among travelers aged ≥18 years was 31% higher during 2015–2019 compared to 2010–2014 (Table 2). Additionally, infections acquired during 2016–2017 (OR = 1.4, 95% CI = 1.3, 1.5) and 2018–2019 (OR = 1.4, 95% CI = 1.3, 1.5) were more likely to be travel-associated than not according to CFRF (Fig. 3; Table S1). During 2015–2019 compared to 2010–2014, *Salmonella* infections increased among travelers to Latin America and the Caribbean (IRR, 1.5 [95% CI, 1.4–1.6]) and decreased among those to Oceania (IRR, 0.4 [95% CI, 0.2–0.9]; Table 2).

Of the 6804 persons with salmonellosis reporting a travel destination, 6679 (98%) reported visiting a single region. While incidence was highest among travelers to Africa (13.1), Asia (9.9) and Latin America and the Caribbean (8.9) during 2010–2014, incidence was highest among travelers to Latin America and the Caribbean (13.3), Asia (11.3) and Africa (10.9) during 2014–2019 (Table 2).

Differences were observed in the distribution of *Salmonella* serotypes by travel-associated versus domestically acquired infections. *Salmonella* Enteritidis was the most common serotype for all travel-associated and domestically acquired infections (Table 3). Serotypes Typhi and Paratyphi A were among the top ten most frequently reported serotypes for travel-associated infections, but these serotypes were rarely observed among domestically acquired infections (ordinally ranked 42 and 75, respectively; Table 3). Among travel-associated infections, 85% of *Salmonella* Typhi and 97% of *Salmonella* Paratyphi A infections were associated with travel to Asia.

### 3.3. Shigella

Shigellosis incidence among travelers aged ≥18 years increased from 2010 through 2019 (Figs. 1 and 2; Tables S1 and S2), with incidence during 2015–2019 being 124% higher compared to 2010–2014 (Table 2). The odds of any illness, regardless of patient age, being travel-associated versus not was significantly lower during 2012–2017 but not 2018–2019 compared to 2010–2011 (Fig. 3; Table S3).

Among all travel-associated *Shigella* infections 2838 (98%) reported travel to a single region, the highest proportion of these persons visited Latin America and the Caribbean (64%). The IR of infections per 100,000 travelers was highest for Africa (12.9), Latin America and the Caribbean (5.1) and Asia (3.4) (Fig. 2).

Of the 1893 speciated travel-associated *Shigella* infections, 40%, 19%, 2% and 1% were caused by *S. sonnei* (n = 759), *S. flexneri* (n = 354), *S. boydii* (n = 41), and *S. dysenteriae* (n = 13).

### 3.4. Counterfactual random forest

The associations identified in the CFRF were consistent with the stratified descriptive summaries (Table 1; Fig. 3; Table S3). For all three pathogens, infections among children under 5 had a greater odds of being domestically acquired. Asian persons were more likely to contract an international travel-associated illness (as opposed to a domestically acquired illness) compared to all other racial groups considered, as were Hispanic compared to non-Hispanic persons. Urban residence was associated with higher odds of travel-associated infection across pathogens. In contrast, indicators of illness severity were generally associated with lower odds of having a travel-associated infection across pathogens, including hospitalization and bloody diarrhea. Persons who had blood as opposed to stool specimens tested were more likely to report travel if they had salmonellosis (but not if they had campylobacteriosis or shigellosis), consistent with practice of ordering blood cultures with clinical concern for TS infection. Fever was associated with travel-associated campylobacteriosis and salmonellosis but not domestically acquired shigellosis. Diagnostic testing characteristics also differed between travel-associated and domestically acquired infections, with persons diagnosed by CIDT being more likely to report travel than persons diagnosed by culture (Table S1).

## 4. Discussion

International travel accounts for a substantial proportion of *Campylobacter*, *Salmonella*, and *Shigella* infections reported in the United States. Like prior studies, we identified that at the species level, *Salmonella* Enteriditis, *Campylobacter jejuni*, and *Shigella sonnei,* were frequently associated with international travel (Amatya et al., 2025; Chanamé-Pinedo et al., 2023; Mughini-Gras et al., 2014). International travel impacted the number of enteric infections reported to FoodNet over the analysis period and mirrored changes in the travel industry through the time period. The odds that an infection was travel-associated increased since 2009, while overall incidence of reported illnesses remained stable, which may reflect increases in international travel, including availability of affordable travel through low-cost air carriers and cruise lines throughout the 2010s [20–22]. A marked decline in reported travel-associated enteric infections observed in 2020 (73% reduction from 2017 to 2019) following implementation of COVID-19–related international travel restrictions, illustrates the contribution of international travel to enteric disease burden in the United States [23,24]. Because FoodNet monitors laboratory-confirmed infections, the data presented here reflect changes in reported incidence and should be interpreted in the context of changes in diagnostics and surveillance during 2010–2019, including increasing use of CIDTs, which have been shown to improve detection of enteric infections.

In this and the previous FoodNet analysis, travel-associated infections differed from domestically acquired infections, occurring disproportionately among working-age adults and urban residents and were less frequently associated with severe outcomes such as hospitalization [7]. The consistency of these patterns across pathogens indicates that travel-associated infections represent a distinct subset of enteric infections that should be interpreted separately from domestically acquired infections in surveillance, clinical, and public health prevention contexts [5,25–27]. However, lower reported severity among travel-associated illnesses should not be interpreted as lower clinical importance. Rather, these differences likely reflect variation in the populations who travel, when and how care is sought, and how infections are detected and reported. For example, the higher odds of mild illness among international travelers may reflect a higher socioeconomic status or a healthy traveler effect, whereby travelers may be healthier and have less chronic disease Travelers may also have access to antibiotics or other preventive therapies provided during a pre-travel clinical consultation, which may reduce illness severity [28]. Lastly, this analysis considered travel-associated infections independent of bacterial resistance; past studies noted that travel-associated antimicrobial resistant (AMR) infections were more likely to result in severe illness compared to non-travel associated illnesses [27]. Follow-up studies are therefore needed to determine how our findings may differ for infections caused by AMR versus antimicrobial susceptible strains of *Campylobacter, Salmonella* and *Shigella*. Regardless, accurate and complete assessment of recent international travel remains critical for interpretation of enteric disease surveillance data and for distinguishing travel-associated from domestically acquired infections in clinical and public health investigations.

Differences in healthcare utilization and diagnostic testing may also influence which travel-associated infections are ultimately identified through surveillance. Recent international travel has been associated with seeking medical care [29], potentially decreasing the likelihood of underascertainment for travel-associated compared to domestically acquired illness. Consistent with this, CFRF identified differences in diagnostic testing practices between travel-associated and domestically acquired infections. CIDTs have become common in outpatient clinical settings, are more sensitive than traditional culture-based methods, and have been associated with improved diagnosis of enteric illnesses [12,19,30,31] Together, these factors may contribute to differences in ascertainment between travel-associated and domestically acquired infections reported through FoodNet. Regardless, accurate and complete assessment of recent international travel remains critical for interpretation of enteric disease surveillance data and for distinguishing travel-associated from domestically acquired infections in clinical and public health investigations.

Pathogen-specific findings further distinguished travel-associated from domestically acquired infections. *Salmonella* Typhi and Paratyphi A were identified almost exclusively as travel-associated illnesses and were rarely domestically acquired, with most reporting travel to Asia. Similar studies conducted in the United States and other countries also linked most *S*. Typhi and Paratyphi A infections to international travel [26,32]. In contrast, *Salmonella* Enteritidis remained common in travel-associated and domestically acquired infections, likely reflecting its global distribution [33]. In this context, findings do underscore that some pathogens are strongly linked to international travel while others circulate broadly in and outside the United States. Knowing the endemicity of enteric pathogens may be helpful when counseling persons about travel-associated risk and recommending prevention measures (i. e., in the case of *S*. Typhi, vaccination).

Incidence of travel-associated enteric infection also varied substantially by destination. Although the largest proportion of travel-associated illnesses was linked to travel to Latin America and the Caribbean followed by Asia, the highest incidence per traveler was observed among persons traveling to Africa, with increasing incidence among travelers to Asia and Latin America and the Caribbean over time. This is consistent with past studies that also noted strong regional differences in illness by travel destination ([34]; Fernandez et al., 2022; [26,35,36]), including the previous analysis that described travel-associated illness reported in the FoodNet catchment during 2004–2009 [6][6]. A previous study on travel-associated antimicrobial resistant *Campylobacter* infection during 2005–2011 reported that Asia (excluding China [21%] and Mexico [20%]) were the destinations with the greatest proportions of illnesses, but the proportion of AMR infections (and thus illness and outcome severity) differed substantially within and between the regions in our analysis [26][26]. These findings indicate that destination-specific patterns of travel-associated enteric illness have persisted over time, highlighting the importance of considering destination-specific risk when clinicians provide pre-travel guidance and post-travel diagnosis. This reinforces that taking a recent international travel history is a pivotal tool for assisting health care practitioners in developing their differential diagnosis of persons presenting with diarrhea [36]. These findings support consideration of destination-specific risk during pre-travel counseling and post-travel clinical evaluation. While general food and water hygiene recommendations remain applicable across destinations, differences in destination-specific risks, antimicrobial resistance patterns, and vaccine-preventable pathogens may warrant additional counseling for selected travelers. For example, the finding that most travel-associated *Salmonella* Typhi and Paratyphi A infections were reported among travelers returning from Asia is consistent with current recommendations that typhoid vaccination be targeted to travelers visiting regions where typhoid fever is endemic. Further studies evaluating region-specific exposures and behaviors could help refine future prevention recommendations.

## 5. Limitations

This analysis has several limitations. Symptom data were missing for a substantial proportion of illnesses; however, missingness for demographic variables was similar for travel-associated and domestically acquired infections, reducing the likelihood of differential bias. Destination-specific incidence estimates relied on SIAT data, which capture international air travel only and exclude land travel, an important limitation for destinations such as Mexico. SIAT also did not include data for travelers to Canada. In addition, incidence rates by destination were limited to travelers aged ≥18 years, although approximately 15% of persons with campylobacteriosis, 26% of salmonellosis, and 25% of shigellosis with travel-associated illness in the present analysis were under 18 years of age. As a result, destination-specific incidence estimates are best suited for comparisons across regions and pathogens rather than as measures of absolute risk, as travel denominator data represents arrivals rather than individual travelers and do not capture duration of travel.

Travel-associated infections in FoodNet are identified only if diagnosed after return to the United States. Ill travelers may not seek care, they may not submit specimens, or infections may not be detected by routine laboratory testing, leading to underestimation of the true burden of travel-associated illness. Previous studies reported etiologic agent identification rates as low as 32% for travel-associated infections, though these findings largely predate the widespread use of CIDTs (Adkins et al., 1990; [37,38]). Conversely, some domestically acquired infections may have been misclassified as travel-associated if diagnosed within the post-travel exposure window, although any resulting overestimation is likely small and would tend to reduce differences between destinations.

Lastly, FoodNet only monitored illnesses caused by eight pathogens. Past studies have shown that *E. coli* pathotypes not surveilled by FoodNet are the leading causes of travel-associated enteric illness [3,4][3,4]. FoodNet does not capture care seeking, or other behavioral data. Additionally, antimicrobial susceptibility data were not studied.

The findings presented here are not intended to be a representative sample of all travel-associated enteric illnesses, just those caused by *Campylobacter, Salmonella* and *Shigella*.

## 6. Conclusion

International travel accounted for a substantial proportion of reported *Campylobacter*, *Salmonella*, and *Shigella* infections in the United States, with the likelihood that an infection is travel-associated increasing over time. Travel-associated infections differed from domestically acquired infections with respect to age, severity, and destination-specific risk, underscoring the importance of collecting travel history during enteric disease surveillance and clinical evaluations. These findings support using targeted prevention efforts among international travelers, including destination-specific counseling and vaccination where appropriate.

## Supplementary Material

Supplemental Material 1

## Figures and Tables

**Table 1:** Demographic and clinical characteristics, by pathogen and travel status — FoodNet, 2010–2019.

	*Campylobacter*				*Salmonella*				*Shigella*			
	Travel-Associated		Domestically Acquired		Travel-Associated		Domestically Acquired		Travel-Associated		Domestically Acquired	
	n/N	%	n/N	%	n/N	%	n/N	%	n/N	%	n/N	%
Total	10015	(16)	51382	(84)	6804	(11)	57019	(89)	2899	(15)	16242	(85)
Age (years)												
0–4	578/10015	(6)	5797/51382	(11)	725/6804	(11)	13581/57019	(24)	292/2899	(10)	3729/16242	(23)
5–17	947/10015	(9)	5372/51382	(10)	1022/6804	(15)	8188/57019	(14)	434/2899	(15)	3978/16242	(24)
18–64	7302/10015	(73)	30985/51382	(60)	4532/6804	(67)	26517/57019	(47)	1881/2899	(65)	7772/16242	(48)
≥65	1188/10015	(12)	9228/51382	(18)	525/6804	(8)	8733/57019	(15)	292/2899	(10)	763/16242	(5)
Median (IQR)	39	(32)	42	(39)	32	(33)	30	(50)	34	(35)	22	(36)
Sex												
Female	4671/10008	(47)	23915/51342	(47)	3555/6800	(52)	30552/56968	(54)	1577/2895	(54)	6627/16213	(41)
Male	5337/10008	(53)	27427/51342	(53)	3245/6800	(48)	26416/56968	(46)	1318/2895	(46)	9586/16213	(59)
Race												
Asian	765/8986	(9)	1945/48086	(4)	889/6256	(14)	2578/52972	(5)	289/2632	(11)	321/14867	(2)
Black	323/8986	(4)	2846/48086	(6)	472/6256	(8)	7728/52972	(15)	308/2632	(12)	4770/14867	(32)
White	7237/8986	(81)	40387/48086	(84)	4403/6256	(70)	39467/52972	(75)	1678/2632	(64)	8456/14867	(57)
Another Race	661/8986	(7)	2908/48086	(6)	492/6256	(8)	3199/52972	(6)	357/2632	(14)	1320/14867	(9)
Ethnicity												
Hispanic	1384/8904	(16)	6086/46872	(13)	828/6189	(13)	6518/52118	(13)	670/2603	(26)	2997/14688	(20)
Non-Hispanic	7520/8904	(84)	40786/46872	(87)	5361/6189	(87)	45600/52118	(87)	1933/2603	(74)	11691/14688	(80)
Rural-Urban Status												
Urban	9423/10015	(94)	40243/51382	(78)	6313/6804	(93)	46530/57019	(82)	2750/2899	(95)	14303/16242	(88)
Rural	592/10015	(6)	11139/51382	(22)	491/6804	(7)	10489/57019	(18)	149/2899	(5)	1939/16242	(12)
Symptoms												
Diarrhea	8428/8608	(98)	42616/43954	(97)	5270/5734	(92)	42148/46124	(91)	2561/2613	(98)	13154/13730	(96)
Fever	5095/8157	(62)	25782/42209	(61)	3656/5346	(68)	28346/43077	(66)	1470/2468	(60)	9124/13287	(69)
Bloody Diarrhea	1912/8123	(24)	13473/42048	(32)	1353/5377	(25)	16182/43626	(37)	687/2407	(29)	5592/13143	(43)
Hospitalizations	753/9920	(8)	11223/51128	(22)	1473/6773	(22)	16633/56703	(29)	344/2845	(12)	3877/16080	(24)
Deaths	2/9667	(.0)	85/50412	(.2)	6/6671	(.1)	199/56342	(.4)	0/2730	()	13/15847	(.1)

**Table 2:** Percent change of incidence rate per 100,000 U S. travelers aged 18 years and over (IR) of travel-associated infections over time, by pathogen and region — FoodNet, 2010–2014 vs 2015–2019.

	2010–2014			2015–2019			% Change in IR
	N	%	IR	N	%	IR	
All U.S. Travelers							
Africa	747223	(3)	-	1048851	(3)	-	-
Asia	5228792	(20)	-	6122663	(17)	-	-
Europe	8367008	(32)	-	11557024	(33)	-	-
Latin America & Caribbean	11503036	(44)	-	15810241	(45)	-	-
North America^a^	151556	(1)	-	93531	()	-	-
Oceania	361529	(1)	-	599439	(1.7)	-	-
Total	26359144	(100)	-	35231749	(100)	-	-
Travel-Associated Infections							
Africa	372	(7)	49.8	606	(6)	57.8	16.1
Asia	1425	(27)	27.3	2448	(25)	40.0	46.7
Europe	855	(16)	10.2	1462	(15)	12.7	23.8
Latin America & Caribbean	2515	(48)	21.9	5227	(53)	33.1	51.2
North America^a^	11	(<1)	7.3	17	(<1)	18.2	150.4
Oceania	45	(1)	12.4	68	(1)	11.3	−8.9
Total	5223	(100)	19.8	9828	(100)	27.9	40.8
*Campylobacter*							
Africa	205	(7)	27.4	330	(6)	31.5	14.7
Asia	775	(26)	14.8	1499	(29)	24.5	65.2
Europe	731	(25)	8.7	1237	(24)	10.7	22.5
Latin America & Caribbean	1191	(41)	10.4	2034	(39)	12.9	24.3
North America^a^	4	(<1)	2.6	9	(<1)	9.6	264.6
Oceania	22	(1)	6.1	47	(1)	7.8	28.8
Total	2928	(100)	11.1	5156	(100)	14.6	31.7
*Salmonella*							
Africa	98	(6)	13.1	114	(4)	10.9	−17.1
Asia	518	(29)	9.9	693	(22)	11.3	14.3
Europe	108	(6)	1.3	166	(5)	1.4	11.3
Latin America & Caribbean	1019	(58)	8.9	2099	(68)	13.3	49.9
North America^a^	7	(<1)	4.6	8	(<1)	8.6	85.2
Oceania	19	(1)	5.3	14	(<1)	2.3	−55.6
Total	1769	(100)	6.7	3094	(100)	8.8	30.9
*Shigella*							
Africa	69	(13)	9.2	162	(10)	15.4	67.3
Asia	132	(25)	2.5	256	(16)	4.2	65.6
Europe	16	(3)	0.2	59	(4)	0.5	167.0
Latin America & Caribbean	305	(58)	2.7	1094	(69)	6.9	161.0
North America^a^	0	(0)	0.0	0	(0)	0.0	0.0
Oceania	4	(1)	1.1	7	(<1)	1.2	5.5
Total	526	(100)	2.0	1578	(100)	4.5	124.4

a Excludes Canada (therefore only Bermuda).

**Table 3:** Top 10 travel-associated *Salmonella* serotypes, by travel status regardless of patient age — FoodNet, 2010–2019.

Serotype^a^	Travel-Associated Infections			Domestically Acquired Infections			*P*-Value
	Rank	n	%	Rank	n	%	
Enteritidis	1	2234	(36)	1	9418	(18)	<0.01*
Typhi	2	525	(8.5)	42	117	(0.2)	<0.01*
Typhimurium	3	354	(5.7)	2	6541	(13)	<0.01*
Newport	4	239	(3.9)	3	5925	(12)	<0.01*
I 4,[5],12:I:-	5	237	(3.8)	5	3176	(6)	<0.01*
Saintpaul	6	193	(3.1)	8	1132	(2.2)	<0.01*
Paratyphi A	7	176	(2.8)	75	46	(0.1)	<0.01*
Infantis	8	147	(2.4)	6	1439	(2.8)	0.1
Javiana	9	108	(1.8)	4	4224	(8.3)	<0.01*
Agona	10	104	(1.7)	19	386	(0.8)	<0.01*
Oranienburg	10	104	(1.7)	13	832	(1.6)	0.7

* Statistically significant (p < 0.05).

a Excludes “Not serotyped” and “Partially Serotyped”.

## Appendix A. Supplementary data

Supplementary data to this article can be found online at https://doi.org/10.1016/j.tmaid.2026.103022.
