# Supplementary material for: Epidemiological comparison of international travel-associated and domestically acquired Campylobacter, Salmonella, and Shigella infections reported to Foodborne Diseases Active Surveillance Network (FoodNet), 2010–2019
Source: Travel Med Infect Dis. Author manuscript; Available in PMC 2026 Sep 21. (PMC13591391; doi:10.1016/j.tmaid.2026.103022)
Supplement: Supplementary file 1 [file NIHMS2210405-supplement-Supplemental_Material_1.docx]

Figure S1: Incidence rate (per 100,000 U.S. travelers aged 18 years and over) of travel-associated campylobacteriosis, by region — FoodNet, 2010–2019

Figure S2: Incidence rate (per 100,000 U.S. travelers aged 18 years and over) of travel-associated salmonellosis, by region — FoodNet, 2010–2019

Figure S3: Incidence rate (per 100,000 U.S. travelers aged 18 years and over) of travel-associated shigellosis, by region — FoodNet, 2010–2019

Table S1: Incidence rate (per 100,00 U.S. travelers aged 18 years and over) of travel-associated infections, by pathogen and year — Foodborne Diseases Active Surveillance Network (FoodNet), 2010–2019

|  | U.S. Travelers |  | FoodNet Travelers | |  | *Campylobacter* | |  | *Salmonella* | |  | *Shigella* | |
| --- | --- | --- | --- | --- | --- | --- | --- | --- | --- | --- | --- | --- | --- |
| Year | N |  | n | IR |  | n | IR |  | n | IR |  | n | IR |
| 2010 | 4723298 |  | 1012 | 21.4 |  | 549 | 11.6 |  | 347 | 7.3 |  | 116 | 2.5 |
| 2011 | 5063674 |  | 971 | 19.2 |  | 548 | 10.8 |  | 331 | 6.5 |  | 92 | 1.8 |
| 2012 | 5622584 |  | 1113 | 19.8 |  | 607 | 10.8 |  | 397 | 7.1 |  | 109 | 1.9 |
| 2013 | 6124065 |  | 981 | 16.0 |  | 563 | 9.2 |  | 333 | 5.4 |  | 85 | 1.4 |
| 2014 | 5418225 |  | 1146 | 21.2 |  | 661 | 12.2 |  | 361 | 6.7 |  | 124 | 2.3 |
| 2015 | 6341740 |  | 1406 | 22.2 |  | 707 | 11.1 |  | 531 | 8.4 |  | 168 | 2.6 |
| 2016 | 6659244 |  | 1720 | 25.8 |  | 904 | 13.6 |  | 570 | 8.6 |  | 246 | 3.7 |
| 2017 | 7229229 |  | 2011 | 27.8 |  | 1039 | 14.4 |  | 690 | 9.5 |  | 282 | 3.9 |
| 2018 | 7617840 |  | 2239 | 29.4 |  | 1226 | 16.1 |  | 609 | 8.0 |  | 404 | 5.3 |
| 2019 | 8001132 |  | 2452 | 30.6 |  | 1280 | 16.0 |  | 694 | 8.7 |  | 478 | 6.0 |
| Total | 62801031 |  | 15051 |  |  | 8084 |  |  | 4863 |  |  | 2104 |  |

Table S2: Incidence rate (per 100,00 U.S. population aged 18 years and over) of infections, by pathogen and year — FoodNet, 2010–2019

|  | U.S. Population |  | FN Cases | |  | *Campylobacter* | |  | *Salmonella* | |  | *Shigella* | |
| --- | --- | --- | --- | --- | --- | --- | --- | --- | --- | --- | --- | --- | --- |
| Year | N |  | n | IR |  | n | IR |  | n | IR |  | n | IR |
| 2010 | 47143646 |  | 16428 | 34.8 |  | 6375 | 13.5 |  | 8276 | 17.6 |  | 1777 | 3.8 |
| 2011 | 47517711 |  | 16141 | 34.0 |  | 6785 | 14.3 |  | 7815 | 16.4 |  | 1541 | 3.2 |
| 2012 | 47886362 |  | 17834 | 37.2 |  | 7695 | 16.1 |  | 7952 | 16.6 |  | 2187 | 4.6 |
| 2013 | 48202808 |  | 17595 | 36.5 |  | 7694 | 16.0 |  | 7390 | 15.3 |  | 2511 | 5.2 |
| 2014 | 48556095 |  | 18126 | 37.3 |  | 7663 | 15.8 |  | 7558 | 15.6 |  | 2905 | 6.0 |
| 2015 | 48926729 |  | 19814 | 40.5 |  | 8494 | 17.4 |  | 8144 | 16.6 |  | 3176 | 6.5 |
| 2016 | 49302075 |  | 19780 | 40.1 |  | 8630 | 17.5 |  | 8214 | 16.7 |  | 2936 | 6.0 |
| 2017 | 49638765 |  | 19597 | 39.5 |  | 9516 | 19.2 |  | 7941 | 16.0 |  | 2140 | 4.3 |
| 2018 | 49940943 |  | 21426 | 42.9 |  | 9832 | 19.7 |  | 9139 | 18.3 |  | 2455 | 4.9 |
| 2019 | 50214837 |  | 20823 | 41.5 |  | 9799 | 19.5 |  | 8596 | 17.1 |  | 2428 | 4.8 |
| Total | 487329971 |  | 187564 |  |  | 82483 |  |  | 81025 |  |  | 24056 |  |

Table S3: Results of the counterfactual random forest where the outcome was if the illness was domestically acquired (reference) or travel-associated.

|  | | | Odds Ratio | 95% Confidence Interval | | *P*-Value |
| --- | --- | --- | --- | --- | --- | --- |
|  |  |  |  | Lower | Upper |  |
| Campylobacteriosis | | | | | | |
|  | Age (Yrs; Ref= Under 5 years) | | | | | |
|  |  | >64 | 1.83 | 1.65 | 2.02 | < 0.001 |
|  |  | 18 –64 | 2.42 | 2.21 | 2.65 | < 0.001 |
|  |  | 5–17 | 2.06 | 1.85 | 2.30 | < 0.001 |
|  | Ethnicity (Ref=Hispanic) | | | | | |
|  |  | Not Hispanic | 0.71 | 0.67 | 0.75 | < 0.001 |
|  | Race (Ref=Asian) | | | | | |
|  |  | Alaskan Native or American Indian | 0.43 | 0.34 | 0.54 | < 0.001 |
|  |  | Another Race | 0.87 | 0.77 | 0.99 | 0.032 |
|  |  | Black or African American | 0.34 | 0.30 | 0.39 | < 0.001 |
|  |  | Multiple Races | 0.54 | 0.43 | 0.69 | < 0.001 |
|  |  | Native Hawaiian or Pacific Islander | 0.46 | 0.28 | 0.75 | 0.002 |
|  |  | White | 0.46 | 0.42 | 0.50 | < 0.001 |
|  | Region (Ref=Mid-Atlantic) | | | | | |
|  |  | Northeast | 0.81 | 0.76 | 0.87 | < 0.001 |
|  |  | Southeast | 0.76 | 0.71 | 0.81 | < 0.001 |
|  |  | Southwest | 0.90 | 0.84 | 0.97 | 0.003 |
|  |  | West | 0.94 | 0.88 | 1.00 | 0.064 |
|  | Season (Ref=Autumn) | | | | | |
|  |  | Spring | 1.28 | 1.20 | 1.37 | < 0.001 |
|  |  | Summer | 1.00 | 0.94 | 1.06 | 0.964 |
|  |  | Winter | 1.44 | 1.35 | 1.55 | < 0.001 |
|  | Sex (Ref=Female) | | 0.98 | 0.94 | 1.02 | 0.325 |
|  |  | Male |  |  |  |  |
|  | Specimen (Ref=Stool) | | | | | |
|  |  | Blood | 0.60 | 0.48 | 0.75 | < 0.001 |
|  |  | Other | 0.90 | 0.74 | 1.11 | 0.345 |
|  | Symptoms and Outcomes | | | | | |
|  |  | Bloody Diarrhea (Ref=No Bloody Diarrhea) | 0.72 | 0.69 | 0.76 | < 0.001 |
|  |  | Diarrhea (Ref=No Diarrhea) | 0.89 | 0.78 | 1.01 | 0.071 |
|  |  | Fever (Ref= No Fever) | 1.09 | 1.04 | 1.14 | 0.001 |
|  |  | Hospitalized (Ref=Not Hospitalized) | 0.38 | 0.36 | 0.41 | < 0.001 |
|  | Test Type [Ref=Culture-Based (Cx)] | | | | | |
|  |  | Culture Independent Diagnostic Test (CIDT) Only | 1.16 | 1.09 | 1.23 | < 0.001 |
|  |  | CIDT with Positive Reflex Cx | 0.94 | 0.88 | 1.02 | 0.120 |
|  |  | CIDT with Negative Reflex Cx | 1.08 | 1.01 | 1.15 | 0.020 |
|  | Urbanicity (Ref=Rural) | |  |  |  |  |
|  |  | Urban | 3.17 | 2.94 | 3.42 | < 0.001 |
|  | Year | | | | | |
|  |  | 2012–2013 | 0.96 | 0.88 | 1.04 | 0.291 |
|  |  | 2014–2015 | 1.02 | 0.94 | 1.10 | 0.699 |
|  |  | 2016–2017 | 1.11 | 1.03 | 1.19 | 0.007 |
|  |  | 2018–2019 | 1.31 | 1.22 | 1.41 | < 0.001 |
| Salmonellosis | | | | | | |
|  | Age (Yrs; Ref= Under 5 years) | | | | | |
|  |  | >64 | 1.35 | 1.22 | 1.48 | < 0.001 |
|  |  | 18-64 | 2.19 | 2.04 | 2.35 | < 0.001 |
|  |  | 5– 17 | 1.85 | 1.69 | 2.02 | < 0.001 |
|  | Ethnicity (Ref=Hispanic) | | | | | |
|  |  | Not Hispanic | 0.85 | 0.79 | 0.92 | < 0.001 |
|  | Race (Ref=Asian) | | | | | |
|  |  | Alaskan Native or American Indian | 0.52 | 0.40 | 0.67 | < 0.001 |
|  |  | Another Race | 0.79 | 0.68 | 0.90 | < 0.001 |
|  |  | Black or African American | 0.31 | 0.27 | 0.34 | < 0.001 |
|  |  | Multiple Races | 0.51 | 0.41 | 0.64 | < 0.001 |
|  |  | Native Hawaiian or Pacific Islander | 0.81 | 0.52 | 1.25 | 0.344 |
|  |  | White | 0.42 | 0.39 | 0.46 | < 0.001 |
|  | Region (Ref=Mid-Atlantic) | | | | | |
|  |  | Northeast | 1.08 | 0.99 | 1.18 | 0.077 |
|  |  | Southeast | 0.54 | 0.50 | 0.58 | < 0.001 |
|  |  | Southwest | 1.07 | 0.97 | 1.18 | 0.155 |
|  |  | West | 1.05 | 0.96 | 1.15 | 0.252 |
|  | Season (Ref=Autumn) | | | | | |
|  |  | Spring | 1.32 | 1.23 | 1.43 | < 0.001 |
|  |  | Summer | 0.89 | 0.83 | 0.96 | 0.002 |
|  |  | Winter | 1.52 | 1.40 | 1.65 | < 0.001 |
|  | Sex (Ref=Female) | | | | | |
|  |  | Male | 1.06 | 1.01 | 1.11 | 0.021 |
|  | Specimen (Ref=Stool) | | | | | |
|  |  | Blood | 1.84 | 1.69 | 2.00 | < 0.001 |
|  |  | Other | 0.62 | 0.55 | 0.69 | < 0.001 |
|  | Symptoms and Outcomes | | | | | |
|  |  | Bloody Diarrhea (Ref=No Bloody Diarrhea) | 0.65 | 0.61 | 0.69 | < 0.001 |
|  |  | Diarrhea (Ref=No Diarrhea) | 0.94 | 0.86 | 1.04 | 0.228 |
|  |  | Fever (Ref= No Fever) | 1.19 | 1.12 | 1.26 | < 0.001 |
|  |  | Hospitalized (Ref=Not Hospitalized) | 0.57 | 0.54 | 0.61 | < 0.001 |
|  | Test Type [Ref=Culture-Based (Cx)] | | | | | |
|  |  | Culture Independent Diagnostic Test (CIDT) Only | 1.52 | 1.34 | 1.72 | < 0.001 |
|  |  | CIDT with Positive Reflex Cx | 1.69 | 1.44 | 1.98 | < 0.001 |
|  |  | CIDT with Negative Reflex Cx | 1.37 | 1.27 | 1.47 | < 0.001 |
|  | Urbanicity (Ref=Rural) | | | | | |
|  |  | Urban | 1.97 | 1.81 | 2.13 | < 0.001 |
|  | Year | | | | | |
|  |  | 2012–2013 | 0.93 | 0.85 | 1.01 | 0.099 |
|  |  | 2014–2015 | 1.08 | 0.99 | 1.17 | 0.067 |
|  |  | 2016–2017 | 1.37 | 1.27 | 1.48 | < 0.001 |
|  |  | 2018–2019 | 1.38 | 1.28 | 1.49 | < 0.001 |
| Shigellosis | | | | | | |
|  | Age (Yrs; Ref= Under 5 years) | | | | | |
|  |  | >64 | 3.13 | 2.66 | 3.69 | < 0.001 |
|  |  | 18–64 | 1.72 | 1.54 | 1.92 | < 0.001 |
|  |  | 5–17 | 1.43 | 1.26 | 1.63 | < 0.001 |
|  | Ethnicity (Ref=Hispanic) | | | | | |
|  |  | Not Hispanic | 0.87 | 0.79 | 0.96 | 0.005 |
|  | Race (Ref=Asian) | | | | | |
|  |  | Alaskan Native or American Indian | 0.24 | 0.16 | 0.37 | < 0.001 |
|  |  | Another Race | 0.47 | 0.38 | 0.58 | < 0.001 |
|  |  | Black or African American | 0.27 | 0.23 | 0.33 | < 0.001 |
|  |  | Multiple Races | 0.22 | 0.14 | 0.33 | < 0.001 |
|  |  | Native Hawaiian or Pacific Islander | 0.18 | 0.07 | 0.45 | < 0.001 |
|  |  | White | 0.30 | 0.26 | 0.36 | < 0.001 |
|  | Region (Ref=Mid-Atlantic) | | | | | |
|  |  | Northeast | 1.50 | 1.28 | 1.77 | < 0.001 |
|  |  | Southeast | 0.50 | 0.44 | 0.56 | < 0.001 |
|  |  | Southwest | 1.36 | 1.17 | 1.58 | < 0.001 |
|  |  | West | 1.67 | 1.47 | 1.90 | < 0.001 |
|  | Season (Ref=Autumn) | | | | | |
|  |  | Spring | 1.20 | 1.06 | 1.36 | 0.003 |
|  |  | Summer | 1.41 | 1.25 | 1.58 | < 0.001 |
|  |  | Winter | 1.27 | 1.12 | 1.44 | < 0.001 |
|  | Sex (Ref=Female) | | | | | |
|  |  | Male | 0.53 | 0.49 | 0.57 | < 0.001 |
|  | Specimen (Ref=Stool) | | | | | |
|  |  | Blood | 0.43 | 0.22 | 0.82 | 0.011 |
|  |  | Other | 0.41 | 0.30 | 0.57 | < 0.001 |
|  | Symptoms and Outcomes | | | | | |
|  |  | Bloody Diarrhea (Ref=No Bloody Diarrhea) | 0.74 | 0.67 | 0.81 | < 0.001 |
|  |  | Diarrhea (Ref=No Diarrhea) | 1.34 | 1.06 | 1.71 | 0.015 |
|  |  | Fever (Ref= No Fever) | 0.87 | 0.80 | 0.95 | 0.003 |
|  |  | Hospitalized (Ref=Not Hospitalized) | 0.58 | 0.52 | 0.65 | < 0.001 |
|  | Test Type [Ref=Culture-Based (Cx)] | | | | | |
|  |  | Culture Independent Diagnostic Test (CIDT) Only | 2.20 | 1.94 | 2.49 | < 0.001 |
|  |  | CIDT with Positive Reflex Cx | 3.01 | 2.68 | 3.37 | < 0.001 |
|  |  | CIDT with Negative Reflex Cx | 1.26 | 1.12 | 1.43 | < 0.001 |
|  | Urbanicity (Ref=Rural) | | | | | |
|  |  | Urban | 1.05 | 0.93 | 1.19 | 0.436 |
|  | Year | | | | | |
|  |  | 2012–2013 | 0.58 | 0.50 | 0.67 | < 0.001 |
|  |  | 2014–2015 | 0.61 | 0.53 | 0.70 | < 0.001 |
|  |  | 2016–2017 | 0.76 | 0.66 | 0.86 | < 0.001 |
|  |  | 2018–2019 | 1.13 | 0.99 | 1.28 | 0.063 |
